# CDC25B Is a Prognostic Biomarker Associated With Immune Infiltration and Drug Sensitivity in Hepatocellular Carcinoma

**DOI:** 10.1155/2024/8922878

**Published:** 2024-09-28

**Authors:** Zixiang Huang, Liangzhi Xu, Zhengqiang Wu, Xiaofeng Xiong, Linfei Luo, Zhili Wen

**Affiliations:** ^1^ Department of Gastroenterology The Second Affiliated Hospital of Jiangxi Medical College Nanchang University, Nanchang, China; ^2^ Department of Hepatobiliary Surgery Ezhou Central Hospital, Ezhou, Hubei, China

**Keywords:** biomarker, cancer immunotherapy, cell division cycle 25B, hepatocellular carcinoma, immune activation

## Abstract

Cell division cycle 25B (CDC25B), a member of the CDC25 phosphatase family, plays a key role in cell cycle regulation. Studies have suggested its carcinogenic potential in various cancers, but the role of CDC25B in the development of hepatocellular carcinoma (HCC) remains poorly understood. The aim of this study was to clarify the role of CDC25B in HCC using bioinformatics and experiments. CDC25B expression data of HCC cancer tissues and paracancerous normal samples were obtained from The Cancer Gene Atlas (TCGA) and Gene Expression Omnibus (GEO) databases, and the relationship between CDC25B expression and the prognosis and degree of tumor differentiation of HCC patients was analyzed. CDC25B expression was verified in clinical HCC tissue samples using fluorescence quantitative polymerase chain reaction (q-PCR) and protein immunoblotting (Western blot). Gene set enrichment analysis (GSEA) was used to identify signaling pathways enriched in CDC25B expression, and differential genes (DEGs) were used to screen out coexpressed hub genes and construct protein-protein interaction (PPI) networks. 5-Ethynyl-2′-deoxyuridine (EDU) staining was used to compare the proliferation and differentiation ability of the HCC cell line (HCC-LM3) after knockdown of CDC25B. Finally, we investigated the mutation of CDC25B in HCC and the relationship between CDC25B expression and tumor cell infiltration of lymphocytes and some immune checkpoints as well as drug sensitivity. CDC25B was overexpressed in HCC tissues and correlated with poor prognosis and the degree of tumor differentiation in patients with HCC. The GSEA and PPI networks together revealed significantly upregulated signaling pathways, as well as functions, associated with the development of HCC when CDC25B was overexpressed. The EDU assay demonstrated that the ability of cells to differentiate value addedly was markedly reduced following the downregulation of CDC25B expression in HCC-LM3s. CDC25B was also involved in the formation of the tumor microenvironment (TME) and immune processes in HCC, and the high expression of CDC25B made patients less sensitive to some drugs. CDC25B can be used as a biomarker and immunotherapeutic target for poor prognosis and partial drug sensitivity in HCC, providing new ideas for HCC treatment.

## 1. Introduction

HCC is one of the most common malignant tumors worldwide, and its occurrence is associated with a variety of factors, including chronic viral hepatitis (especially hepatitis B and C viruses), alcoholic liver disease, nonalcoholic fatty liver disease, as well as environmental and genetic factors [1, 2]. The prognosis for HCC is usually poor, in part because of the often advanced stage at diagnosis and its relative resistance to chemotherapy and radiation, and because biological markers such as alpha-fetoprotein, which are widely used in clinical practice, do not accurately reflect the prognosis of patients [3]. Therefore, it is important to identify biomarkers that promote the transformation to malignancy and diagnosis of HCC, as well as relevant therapeutic molecular targets.

The formation of almost all tumors, including hepatocellular carcinoma, originates from the unlimited growth and differentiation of normal cells in the body [4]. The normal cell cycle includes interphase (G phase) and mitosis (M phase). The interphase is further divided into three subphases: the G1 phase, the S phase, and the G2 phase [5].CDC25B as a cell cycle regulatory protein is mainly involved in two processes; firstl, it promotes the transition from G2 to the M phase by removing the inhibitory phosphoryl group on CDK1; secondly, it maintains the activity of CDK1 during the later stages of M to complete cell division [6]. Due to the key role of CDC25B in cell cycle regulation mentioned above, it is aberrant expression or dysfunction is likely to lead to an uncontrolled cell cycle and thus tumorigenesis. However, how CDC25B promotes the development of HCC, in what ways it affects HCC patients, and the molecular mechanisms of its action remain to be elucidated.

This study is aimed at investigating the critical role of CDC25B in HCC patients and its potential molecular mechanisms.

## 2. Results

### 2.1. CDC25B Is Differentially Expressed in Hepatocellular Carcinoma Tissues and Paracarcinoma Tissues

**Figure 1:**
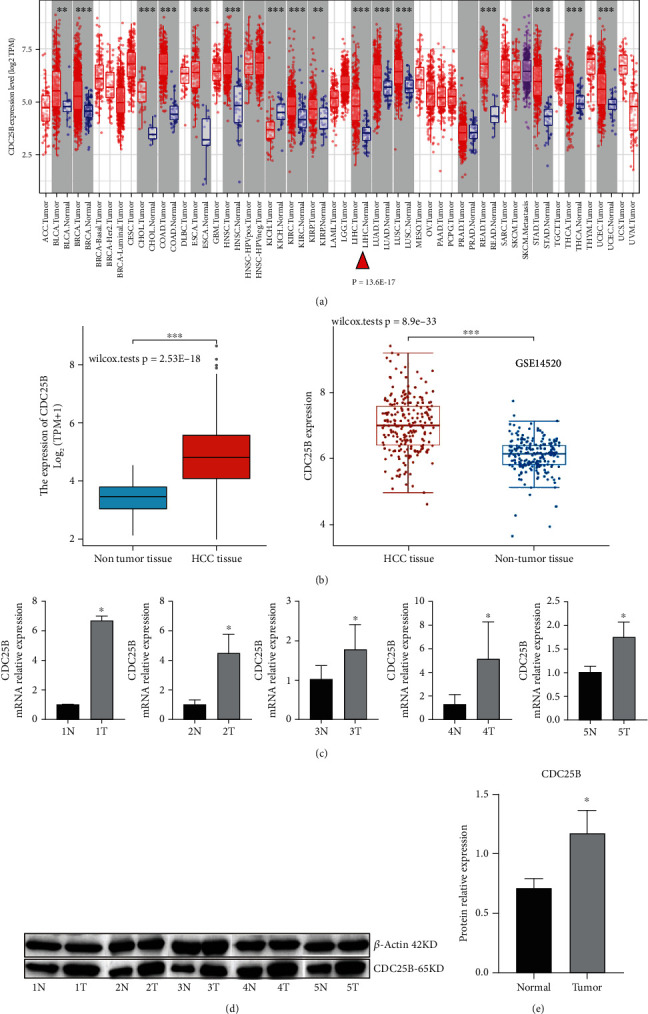
CDC25B is overexpressed in HCC. (a) CDC25B expression in pan-cancer was analyzed using the TIMER database, and CDC25B was differentially expressed in cancer tissues and paracancerous tissues of HCC patients. (b) CDC25B was overexpressed in HCC patients' cancer tissues compared with paracancerous tissues in the TCGA database and GEO database. (c) q-PCR results showed that CDC25B was overexpressed in cancer tissues compared with paracancerous tissues in five cases of HCC. (d) Western blot showed that CDC25B was overexpressed in 5 HCC cancer tissues compared with paracancerous tissues. (e) Histogram of the difference in CDC25B protein expression in five cases of cancer tissues and paracancerous tissues. (^∗^*p* < 0.05, ^∗∗∗^*p* < 0.001).

Analysis of RNA sequencing data from HCC patients downloaded from the GEO database (https://www.ncbi.nlm.nih.gov/geo/) and from the TCGA database (https://portal.gdc.com) showed that CDC25B was differentially expressed in HCC cancer tissues and paracancerous tissues (Figures 1(b) and 1(c)). TIMER database analysis showed that CDC25B was also differentially expressed in a variety of other tumors (Figure 1(a)).

### 2.2. Differential Expression of CDC25B in Hepatocellular Carcinoma Tissues and Paracarcinoma Tissues Was Verified by Quantitative Polymerase Chain Reaction (q-PCR) and Western Blot at RNA and Protein Levels, Respectively

Real-time q-PCR and protein immunoblotting (Western blot) confirmed that CDC25B expression was significantly upregulated in cancer tissues compared with paracancerous tissues in clinical samples (Figures 1(d), 1(e), and 1(f)). So interfering with the expression and activity of CDC25B may be a potential method to intervene in the progression of HCC.

### 2.3. CDC25B Overexpression Is Associated With Poorer Survival and Higher Tumor Grade in HCC Patients

**Figure 2:**
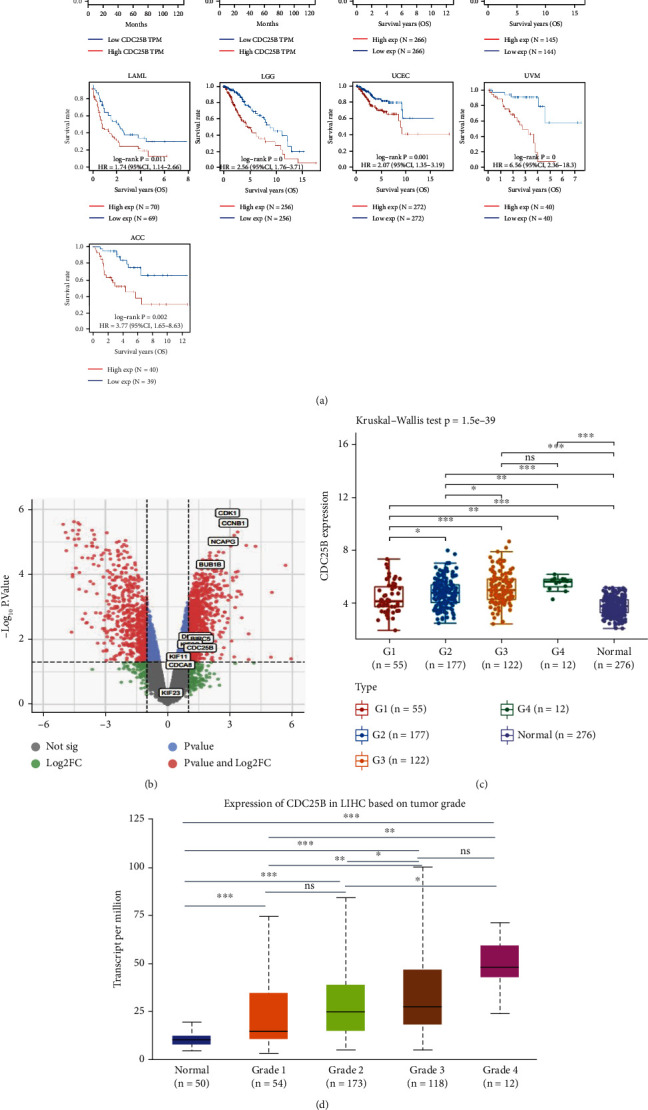
Survival curves of high and low CDC25B expression groups in HCC and pan-cancer, and positive correlation between CDC25B expression and the degree of tumor differentiation in HCC patients. (a) The final survival and disease-free survival of HCC patients in the CDC25B low expression group was better than that in the high expression group, in ACC, KIRC, KIRP, LAML, LGG, UCEC, and UVM tumors. (b) Differential gene volcano plots of cancer tissues and paracancerous tissues of HCC patients in the TCGA database (labeled genes are CDC25B and hub genes). (c) Relationship between CDC25B and the degree of tumor differentiation in HCC patients in the GEO database (normal vs. Grade 1, normal vs. Grade 2, normal vs. Grade 3, normal vs. Grade 4, Grade 1 vs. Grade 3; *p* < 0.001; Grade 1 vs. Grade 4, Grade 2 vs. Grade 4; *p* < 0.01; Grade 1 vs. Grade 2, Grade 2 vs. Grade 3; *p* < 0.05; Grade3 vs. Grade 4; no significance) (d) Relationship between CDC25B and the degree of tumor differentiation in HCC patients in TCGA database. Normal versus Grade 1, normal versus Grade 2, normal versus Grade 3, normal versus Grade 4 *p* < 0.001; Grade 1 versus Grade 3, Grade 1 versus Grade 4; *p* < 0.01; Grade 2 versus Grade 3, Grade 2 versus Grade 4; *p* < 0.05; Grade 1 versus Grade 2, Grade 3 versus Grade 4; no significance (^∗^*p* < 0.05; ^∗∗^*p* < 0.01; ^∗∗∗^*p* < 0.001).

Survival analysis was performed, and survival curves were plotted using the Kaplan–Meier method. We found that among a variety of tumors including HCC, all of them had a better clinical prognosis in the CDC25B low expression group (Figure 2(a)). In addition, we graded HCC patients from the GEO database and the TCGA database according to the degree of tumor differentiation, where Grades 1–4 represent highly differentiated, moderately differentiated, poorly differentiated, and undifferentiated, respectively. The results showed that tumor tissues at all levels of differentiation exhibited differences in CDC25B expression compared with normal tissues (Figures 2(c) and 2(d)). It was suggested that high CDC25B expression was associated with higher tumor differentiation in patients.

### 2.4. Single Gene Set Enrichment Analysis (GSEA) Identifies Pathways Significantly Upregulated and Enriched Upon High CDC25B Expression in HCC

**Figure 3:**
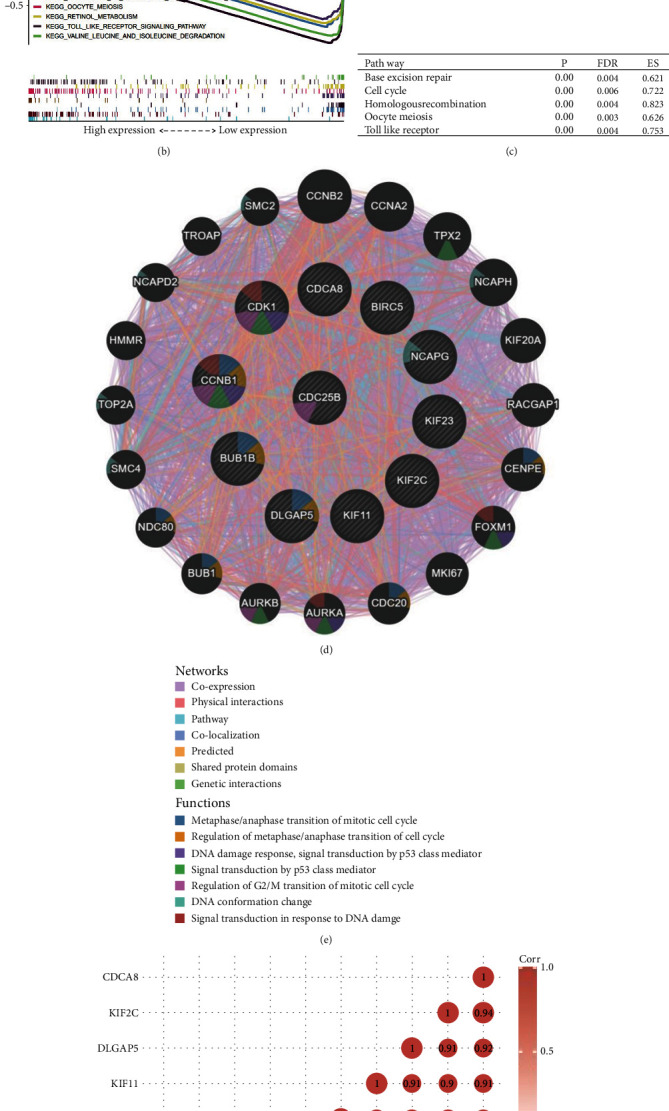
PPI network and gene correlation analysis of GSEA signaling pathway map and hub gene constructs. (a) Signaling pathways enriched in the CDC25B high expression group in GSEA for base excision repair, cell cycle, homologous recombination, meiosis, and Toll-like receptor signaling pathways. (b) Map of the most significantly up and downregulated signaling pathways enriched in GSEA. (c) *p* values and FDR and ES values of significantly upregulated pathways. (d, e) Diagrams of PPI networks constructed by hub genes, whose main functions are G2/M transition and regulation of mitotic cell cycle, cell division, DNA conformational changes, and DNA damage repair mediated by the P53 cascade signaling pathway. (f) Heatmap of the correlation between hub genes. (g) Correlation between CDC25B and the hexameric complex of dehelicase.

GSEA enrichment analysis identified pathways that were significantly upregulated when CDC25B was highly expressed (Figure 3(a)), and the functions of the significantly upregulated pathways were base excision repair, cell cycle, homologous recombination, meiosis, and Toll-like receptor signaling pathways. These upregulated signaling pathways affect cell proliferation and differentiation as well as drug sensitivity. The confluence of the 10 most significantly up and downregulated pathways enriched by GSEA enrichment analysis was also plotted (Figure 3(b)). The corresponding *p* values, enrichment scores, and false discovery rates (FDR) were calculated (Figure 3(c)).

### 2.5. PPI Network and Hub Gene Genes Further Reveal the Biological Function of CDC25B

**Figure 4:**
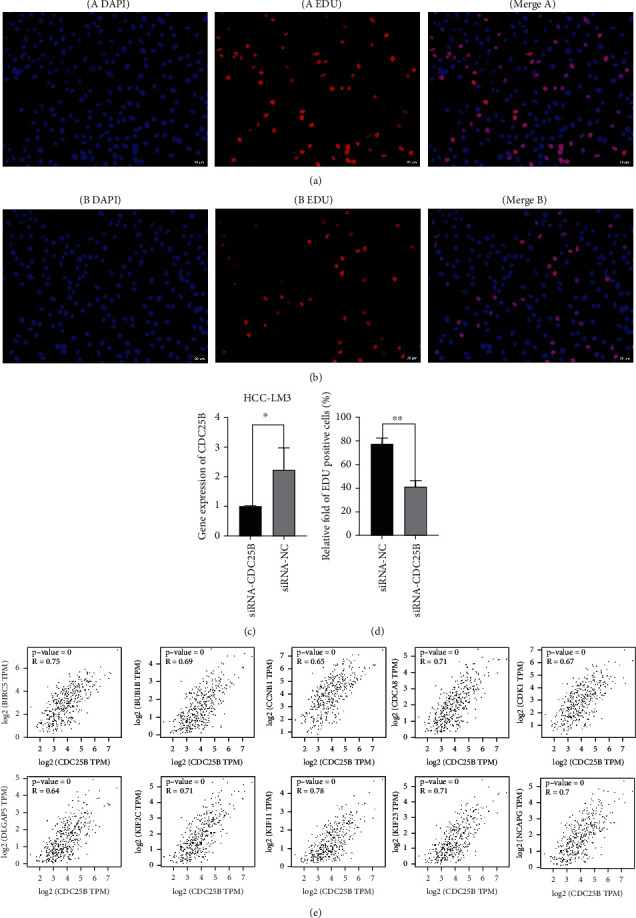
Fluorogram of the efficiency of siRNA knockdown of HCC-LM3 cell line and the proliferative capacity of the cell line detected by EDU staining method. (a, b) Significantly more cells in the DNA synthesis phase in the cell lines without knockdown of CDC25B than after knockdown of CDC25B. (c) siRNA successfully knocked down CDC25B in the HCC-LM3 cell line. (d) Histogram of the difference in the proportion of EDU-positive cells between the knockdown and nonknockdown groups. (e) Scatter plot of the correlation between hub genes and CDC25B (^∗^*p* < 0.05; ^∗∗^*p* < 0.01).

To further understand the regulatory mechanism of CDC25B and discover new regulatory molecules. We analyzed cancer tissue and paracarcinoma tissue differential gene expression (DEG) in HCC samples from the TCGA database (Figure 2(b)). Then, 10 coexpressed hub genes were screened out from 141 DEGs strongly correlated (correlation coefficient > 0.6) with CDC25B using the string database and the Cytohubba plug-in of Cytoscape software, which were CCNB1, BUB1B, BIRC5, KIF23, DLGAP5, CDK1, KIF11, NCAPG, KIF2C, and CDCA8. The coexpression network and correlation among the hub genes constructed from these 10 top-ranked hub genes and CDC25B are shown in Figures 3(d) and 3(f)). These genes are mainly responsible for the G2/M transition and regulation of the mitotic cell cycle, cell division, DNA conformational changes, and DNA damage repair mediated by the P53 cascade signaling pathway (Figure 3(e)). The scatter plot of the correlation between the hub genes and CDC25B is shown (Figure 4(e)). In addition, we found that CDC25B was strongly correlated with the deconjugation enzyme hexameric complex (minichromosome maintenance MCM) (Figure 3(g)).

### 2.6. Reduced Cell Line Proliferation and Differentiation Capacity After Knockdown of CDC25B in HCC-LM3 Cell Line

The HCC-LM3 cell line CDC25B was successfully knocked down using siRNA (Figure 4(c)). EDU assay showed that the number of cells in the cytosolic phase was significantly reduced after knocking down the CDC25B gene of the cell line using siRNA (Figures 4(a) and 4(b)), and the bar graph of the difference in the number of cells with EDU positivity is shown in Figure 4(d).

### 2.7. Correlation of CDC25B With Levels of Tumor-Infiltrating Lymphocytes and With Tumour Purity

**Figure 5:**
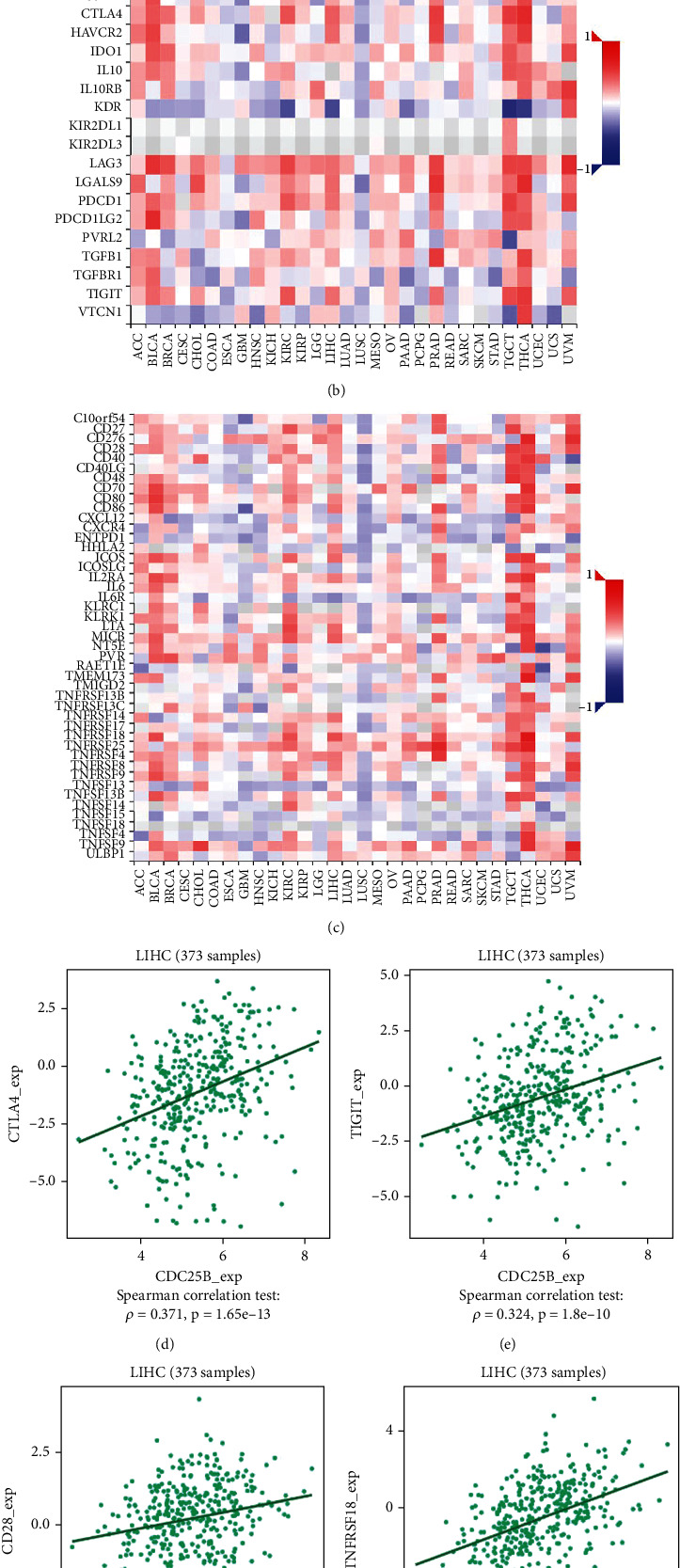
CDC25B positively correlates with immune infiltrating lymphocytes and immune checkpoints (a) CDC25B expression was significantly and positively correlated with the level of immune infiltration (B cells, CD8+ T cells, CD4+ T cells, macrophages, neutrophils, and dendritic cells) in HCC tissues and did not significantly correlate with tumor purity. (b) Heat map of CDC25B correlation with immunosuppressive sites. (c) Heatmap of CDC25B correlation with immunostimulatory sites. (d, e) Scatter plots of CTLA4 and TIGIT correlation with CDC25B expression. (f, g) Scatterplot of CD28 versus TNFRSF18 versus CDC25B expression.

CDC25B expression in HCC cells had a significant correlation with B cells, CD8+ T cells, CD4+ T cells, macrophages, neutrophils, and dendritic cells in tumor cells (T Figure 5(a)). It indicated that CDC25B gene expression was positively correlated with lymphocyte infiltration abundance, tumor purity (TP) is the percentage of tumor cells in a tumor sample, and the results of the study showed that CDC25B expression was not associated with TP.

The correlation of CDC25B expression with certain tumor immune checkpoints suggests that CDC25B participates in tumor immune response.

Using information from the TISIDB database, we investigated the correlation of some immune checkpoints with CDC25B and plotted a heat map (Figures 5(b) and 5(c)). Most of the immune checkpoints, especially CTLA4, TIGIT CD28, and TNFSRF18, were positively correlated with CDC25B (Figures 5(d), 5(e), 5(f), and 5(g)). It suggests that CDC25B may be involved in the regulation of tumor immune response by affecting immune checkpoint activity.

### 2.8. Evaluation of Drug Sensitivity

**Figure 6:**
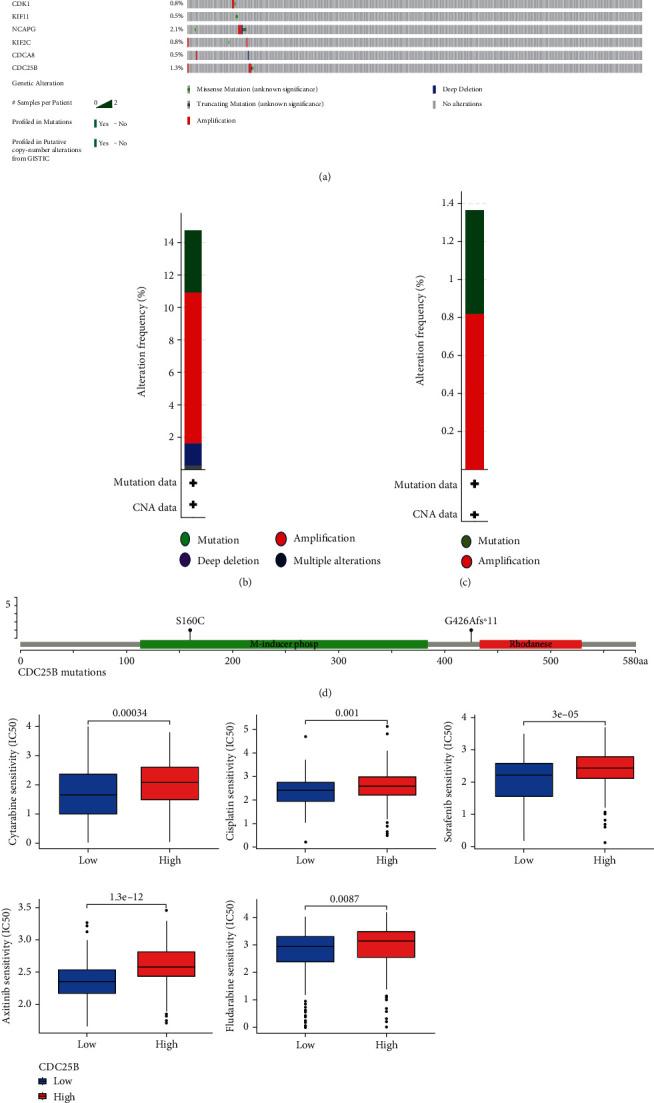
Mutation status of CDC25 and hub genes and the relationship between CDC25B expression and ic50 of selected drugs. (a) Total graph of CDC25B and hub gene mutations in HCC patients in the TCGA database, with 60 of 379 HCC patients (16%) having genetic alterations in 11 key genes. (b, c) Total mutation frequency of the 11 key genes and mutation frequency of the CDC25B gene. (d) Two specific mutations in CDC25B (S160C and G426Afs^∗^11) in HCC patients. (e) Comparison of IC50 between high and low CDC25B expression groups in different HCC therapeutic drugs. (*p* ≤ 0.001).

Cell cycle regulatory factors have been reported to interact with chemotherapy. To further explore the possibility of CDC25B in the individualized treatment of HCC patients, we investigated the relationship between the drug half inhibitory concentration (IC50) of various chemotherapeutic agents for the treatment of HCC and the expression of CDC25B in hepatocellular carcinoma cells, and here, we have attached the IC50s of the clinically common drugs including sorafenib, cytarabine, cisplatin, axitinib, and fludarabine and the relationship of CDC25B expression. The results showed that patients in the low CDC25B expression group were more sensitive to the above drugs than those in the high expression group (Figure 6(e)).

### 2.9. CDC25B Gene-Specific Mutations in HCC Patients

Mutations in genes may be useful in the diagnosis and detection of diseases. For this purpose, we analyzed mutations in CDC25B and hub genes (CCNB1, BUB1B, BIRC5, KIF23, DLGAP5, CDK1, KIF11, NCAPG, KIF2C, and CDCA8) in HCC samples at the cBioPortal database (Figure 6(a)). The frequency of mutations in 11 hub genes is shown (Figures 6(b) and 6(c)). Among them, CDC25B had 2 specific mutations S160C and G426Afs^∗^11 in HCC samples (Figure 6(d)). Due to the low frequency of mutations occurring in the CDC25B gene, there were not enough mutated samples to support the study of the impact of CDC25B mutations on the survival prognosis.

## 3. Discussions

In the study, we found that CDC25B was highly expressed and correlated with poor prognosis and degree of tumor differentiation in HCC patients in both database and HCC clinical samples. In vitro, the HCC-LM3 cell line with knockdown of CDC25B had decreased proliferation, suggesting that high expression of CDC25B enhances the proliferation of hepatocellular carcinoma cells in vitro and tumorigenicity in vivo. The GSEA combined with the PPI network showed that overexpression of CDC25B also led to overactivation of the DNA damage repair response mediated by base excision repair, cell cycle, homologous recombination, mitophagy, Toll-like receptor signaling pathway, and P53 in tumor cells. P53-mediated DNA damage repair response is overactive. The overactivity of these pathways and functions not only promotes cell overproliferation but also affects the sensitivity of HCC patients to some chemotherapeutic agents. In addition, the expression of CDC25B was positively correlated with the abundance of multiple lymphocyte infiltrates and the upregulation of immune checkpoints, and our study supports the idea that CDC25B is a potential biomarker for the prognosis of HCC.

Unlimited cell proliferation and differentiation is a common feature of tumors. The EDU assay showed a significant decrease in the number of cells in the DNA synthesis phase in the HCC-LM3 cell line after the knockdown of CDC25B. CDC25B acts during the G2/M phase transition of cells. It activates cell cycle-dependent kinases (CDKS) (especially CDK1) through dephosphorylation, and CDK1 then binds to cyclinB to form the cyclinB-CDK1 complex to advance cell cycle progression, but this process is only the initial, and full activation of the cyclinB-CDK1 complex requires the action of CDC25C in the nucleus [6, 7]. CDC25B also plays an important role in the process of DNA damage repair, and when intracellular DNA damage occurs, relevant checkpoints (CHK1 and CHK2) respond to regulate CDC25B activity and inhibit cell division until DNA repair is complete [8]. The PPI network showed that CDC25B and hub genes regulate DNA repair damage through the P53 signaling pathway, which is consistent with previous studies. For example, P53 has been shown in many studies to play a role in biological processes such as cell cycle arrest, senescence, DNA repair, and apoptosis; in addition, the P53 signaling pathway is involved in a variety of functions such as autophagy, cellular metabolism, and pathways involved in reactive oxygen species generation [9–12]. It is well known that mitogen-activated protein kinase (MAPK) mediates an important cellular signaling pathway in cells [13, 14]. Its members include the P38MAPK signaling pathway, ERK (extracellular signal-regulated kinase), and JNK (c-JunN-terminal kinase) [15]. It has been reported that the JNK signaling cascade is usually responsible for DNA damage-induced apoptotic responses, whereas the P38MAPK and ERK signaling cascades are mainly responsible for cell proliferation [16]. The P38MAPK signaling pathway participates in the stress response during intracellular DNA damage (ionizing radiation, UV induction, and replication inhibitors) by phosphorylating CDC25B [17]. The MCM complex acts as a key complex molecule for deconjugating enzymes at the onset of the cell cycle, and one of its components, minimal chromosome maintenance complex component 7 (MCM7), influences cell cycle progression through its involvement in the MAPK-CDK1 pathway, whereas CDK1 activity is directly affected by CDC25B [16]. For this reason, we further investigated the correlation between CDC25B and the MCM complex, and the results showed that CDC25B was strongly correlated with the MCM complex. Among them, MCM7, as an important subunit of MCM deconjugating enzyme, is associated with the development of many tumors, including HCC [16]. This functional role allows us to further determine that CDC25B and MCM complex workers coactivate the MAPK pathway, particularly the ERK and p38 signaling cascade to promote cell proliferation and differentiation, which plays an important role in subsequent cancer progression. Unfortunately, correlation analysis between MCM7 and CDC25B expression in hepatocellular carcinoma cell lines or clinical hepatocellular carcinoma tissues was not performed in this study to further validate the important role of MCM7 in the CDC25B-CDK1 axis. We look forward to more such studies in the future to confirm this idea.

In this study, CDC25B was highly expressed in a variety of tumors, including HCC, and correlated with the degree of tumor differentiation in HCC patients [18]. This is consistent with previous studies. Our study found that among the HCC tumor samples from the two databases, all levels exhibited differences in CDC25B expression, except for tumor differentiation at Grades 3 and 4 and Grades 1 and 2 in the TCGA database, which did not show significant differences in CDC25B expression. In conjunction with the in vitro experiments in this study, the knockdown of CDC25B expression in cell lines resulted in a decrease in the proliferative differentiation capacity of the cells, and this suggests that overexpression of CDC25B promotes the overproliferation and differentiation of HCC cells. Previous studies have demonstrated that cell cycle regulation can synergize with chemotherapy in both promotion and inhibition [19, 20].Therefore, we investigated the relationship between CDC25B expression and sensitivity to some drugs as well as immune checkpoints, and we found that in HCC patients with high expression of CDC25B, their sensitivity to cisplatin, sorafenib, cytarabine, fludarabine, and axitinib was significantly reduced. GSEA suggests that the significantly upregulated signaling pathways reveal the mechanism by which high expression of CDC25B affects the efficacy of chemotherapy. The base excision repair signaling pathway is one of the important pathways for cellular DNA damage repair, which is mainly responsible for repairing small base damages in DNA, such as deamination, oxidation, alkylation of deoxyribonucleic acid, or other single-base damages. This pathway could have reduced the risk of oncogenic mutations brought about by DNA damage, and it could have maintained genomic stability. When this signaling pathway is particularly active in the cell, the clinical use of certain chemotherapeutic agents to treat HCC patients becomes less effective. This is because an overactive signaling pathway may repair these damages more efficiently, leading to drug resistance [21]. Homologous recombination signaling pathways are also more effective in repairing chemotherapy- and radiation-induced DNA damage when they are overactive [22]. In particular, cisplatin-sensitive cancer cells may escape the toxic response to the drug by homologous recombination [23]. The meiotic signaling pathway when overactive can lead to tumor cells acquiring the ability to proliferate indefinitely. Long-stranded noncoding RNAs (lncRNAs) have been reported to play especially important roles in the development of HCC, one of which is by influencing cell cycle progression. SNHG16 (small nucleolar RNA host gene 16), a lncRNA, has been reported to be able to not only promote cell proliferation by regulating the CDC25B-CDK1 axis but also to influence the sensitivity of HCC patients to drugs such as cisplatin [24]. Thus, our study further identified CDC25B as a downstream target of SNHG16 affecting the sensitivity of HCC patients to drugs such as cisplatin. In conclusion, our findings on drug sensitivity are very meaningful. In addition, it has been documented that genes regulating the cell cycle can also influence immunotherapy in patients. For example, CDK4, CDK6, and CDK7 can influence immunotherapy efficacy by affecting the tumor microenvironment (TME) [25, 26]. CDK4 and CDK6 inhibitors have now been shown to promote the formation of CD8+ T memory cells, thereby enhancing antitumor immunity [27]. CDK7 inhibitors can improve the efficacy of anti-PD-1 therapy for non-small cell lung cancer (NSCLC) [28].Therefore, we further investigated the relationship between CDC25B and the TME and immune checkpoints. In this study, we found that high expression of CDC25B also caused an increase in the abundance of lymphocytes and overexpression of immune checkpoints within the tumor cells, which creates a proinflammatory and proimmune microenvironment; it causes an overactivation of the TME. GSEA analyses showed that the CDC25B high-expression Toll-like receptor signaling pathway was significantly upregulated in the group. It has been reported that the Toll-like receptor signaling pathway not only plays an active role in suppressing immune surveillance of tumors but also alters the TME by promoting inflammatory responses and facilitating immune cell infiltration as well as tumor angiogenesis [29, 30].The possibility that the upregulation of immune checkpoints may be one of the mechanisms leading to the evasion of HCC cells from the immune system, these factors further explain the poorer prognosis of patients with high CDC25B expression. Immunotherapies targeting immune checkpoints in HCC cells, such as antiprogrammed death protein 1 (PD1) and antiprogrammed death ligand 1 (PDL1) inhibitors, have been available in the past [31, 32]. Our study found that immune checkpoints other than PD1 and PDL1 were positively correlated with CDC25B expression and further demonstrated that CDC25B can be used not only as a prognostic biomarker but also as an immunotherapeutic target. In this study, the frequency of CDC25B-specific mutations in the TCGA database in HCC patients was low, and there were insufficient data to support the study of the effect of the mutations on survival prognosis, but the S160C and G426Afs^∗^11 mutations, the substitution of a serine by a cysteine at position 160 and a glycine at position 426, due to an insertion or a deletion, resulted in changes in the downstream amino acid sequence and cause code-shifting mutations. These mutations may lead to abnormal or early inactivation of CDC25B protein activity, thereby affecting the cell cycle and DNA damage repair response. Overall, understanding the mutation profile of CDC25B can help us to provide new targets for cancer treatment as well as personalized health management and prevention strategies for high-risk populations based on the mutation profile of CDC25B. We look forward to more future studies on the effects of CDC25B mutations in HCC patients.

There have been studies related to CDC25B-selective inhibitors for the treatment of other tumors, such as gastric cancer, ovarian cancer, and nonmelanoma skin cancer [33]. Poor prognosis has been reported in gastric cancer patients with high CDC25B expression [34]. CDC25B-specific inhibitors all inhibited the proliferation and invasion of the above tumor cells [35, 36]. However, the efficacy of CDC25B selective inhibitors in HCC patients has been less studied. In this study, the EDU experiment proved that the proliferation and differentiation ability of HCC cell lines decreased significantly after the knockdown of CDC25B, and drug sensitivity analysis suggested that the overexpression of CDC25B could reduce the sensitivity of some chemotherapeutic drugs, so we judged that CDC25B-selective inhibitors could also benefit HCC patients.

In summary, CDC25B, a key factor involved in cell cycle regulation (mainly G2/M phase), was shown to be overexpressed in HCC tumors and significantly correlated with poor prognosis and degree of tumor differentiation in HCC patients. In vitro, the knockdown of CDC25B significantly inhibited the proliferative capacity of HCC cell lines. A series of signaling pathways overactivated when CDC25B was overexpressed was analyzed by GSEA in conjunction with the PPI network, which could explain the reduced sensitivity of some chemotherapeutic agents when CDC25B was highly expressed. Combined with the existing studies, we can determine that CDC25B promotes the proliferation of HCC cells by participating in the MAPK signaling pathway and mediates DNA damage and repair through the P53 signaling pathway; in addition, the overexpression of CDC25B affects the alteration of the TME and the upregulation of immune checkpoints. Our study reveals the potential of CDC25B as an immunotherapeutic target for HCC.

However, there are some shortcomings in our study, such as the fact that we did not validate the function of CDC25B in a variety of hepatocellular carcinoma cell lines, and we did not validate the results of the biosignature analysis by performing experiments on the signaling pathway mechanisms.

## 4. Conclusion

In conclusion, our results demonstrated the adverse effects of CDC25B on tumor differentiation and prognosis in HCC patients. Overexpression of CDC25B markedly upregulated intracellular signaling pathways regulating the cell cycle, base excision repair, homologous recombination, meiosis, and Toll-like receptor, which promoted unlimited cell proliferation and differentiation, and further affected the sensitivity of HCC patients to some drugs. In addition, high CDC25B expression contributed to the poor prognosis of HCC patients by altering the abundance of lymphocyte infiltration, creating an overactive TME, and upregulating some immune checkpoints, which exacerbated the immune evasion mechanism of the tumor. Our study demonstrated that CDC25B can be used not only as a prognostic biomarker but also as an immunotherapeutic target for HCC, which provides important clues for future research on personalized therapeutic strategies for HCC.

## 5. Materials and Methods

**Figure 7:**
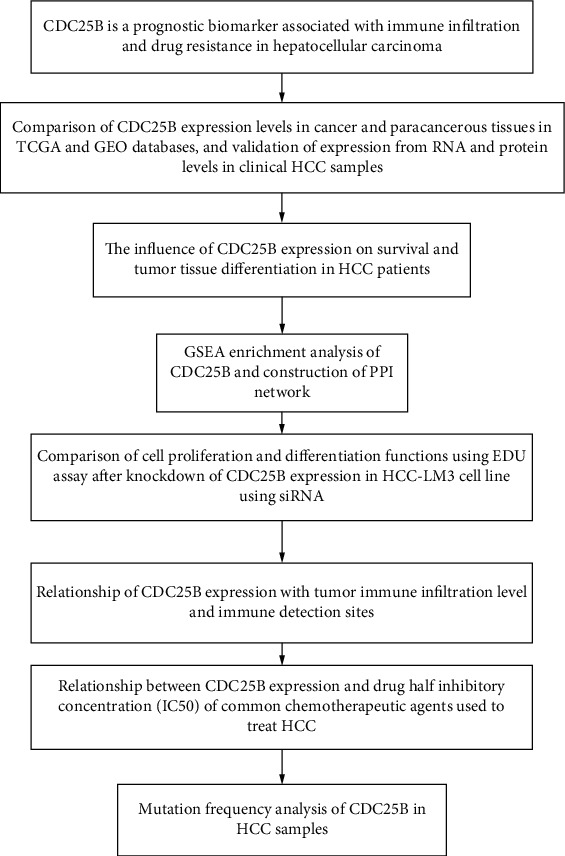
Workflow. The workflow of this study.

The workflow is shown in Figure 7.

### 5.1. Data Sources

RNA sequencing data from HCC patients were downloaded and collated from the GEO database for the GSE14520 dataset (https://www.ncbi.nlm.nih.gov/geo/) and from the TCGA database (https://portal.gdc.com). CDC25B expression in pan-cancer was then analyzed using the TIMER database. Clinical samples were obtained from a total of 10 tissue samples from five patients with hepatocellular carcinoma in the Second Affiliated Hospital of Nanchang University, including five tumor tissue samples and five paracancerous tissue samples, and the samples were stored in a −80°C refrigerator before the experiment.

### 5.2. q-PCR and Western Blot

Differential expression of CDC25B in cancerous and paracancerous tissues in clinical samples was validated using q-PCR and Western blot at the RNA and protein levels, respectively, for a total of 10 clinical HCC samples containing five cancerous and five paracancerous tissues.

### 5.3. Kaplan–Meier Survival Analysis

We investigated the effect of CDC25B expression on overall survival (OS) and disease-free survival (DFS) and the degree of tumor differentiation in HCC patients by gene expression profiling interaction analysis GEPIA (http://gepia.cancer-pku.cn/)database. The expression of CDC25B in pan-cellular carcinomas and the corresponding clinical information were obtained from the TCGA database and the effect of CDC25B expression on pan-cellular carcinoma survival was analyzed. Log-rank test and COX regression analysis were used to compare the survival differences between the CDC25B high-expression group and low-expression group, and *p* values and hazard ratios (HRs) with 95% confidence intervals (CIs) were calculated, with *p* < 0.05 representing a significant difference.

### 5.4. GSEA

To gain a deeper understanding of the biological function of CDC25B in HCC, we downloaded the gene set of HCC patients from the TCGA database and constructed a gene expression matrix, and after screening the target gene CDC25B, the median CDC25B expression in the cohort was ranked as a threshold, and the samples were divided into two groups: the high-expression group and the low-expression group. GSEA was used to analyze the enrichment differences in signaling pathways between the CDC25B high-expression group and low-expression group.

### 5.5. Identification of DEGs and Construction of the PPI Network

The RNA sequencing data of HCC patients from the TCGA library were used to search for DEGs in cancer tissues and paracarcinoma tissues of HCC patients, a gene expression matrix was constructed based on the expression of DEGs, and the correlation between CDC25B gene and DEGs was analyzed by combining with R language software. Coexpression hub genes were screened based on DEGs based on STRING online database and Cytoscape software. Cytohubba plug-in, as an extension module of the Cytoscape platform, provides an efficient strategy for identifying key nodes in biological networks, which integrates 11 different computational algorithms covering four local network characterization-based methods and seven methods based on global network properties. Among these algorithms, maximum clique centrality (MCC), an innovative technique, improves the sensitivity and specificity of the analysis and accurately identifies the hub genes in the network. Finally, a PPI network was constructed using CDC25B cohub genes at the GENEMANIA online site.

### 5.6. Knockdown of CDC25B in the HCC-LM3 Cell Line Using Small Interfering RNAs

CDC25B-specific siRNA, blank control siRNA, and HCC-LM3 cell line were purchased from Gibthai (Guangzhou, China) The sequence of the specific siRNA was siRNA1CDC25B (GCCGGAUCAUUCGAAACGATT), siRNA2CDC25B (GGAAAAGGACCUCGUCAUGTT), and siRNA3CDC25B (GCUCUUACUCUUUCCUAUUTT). These siRNAs were transfected into the HCC-LM3 cell line using transfection reagents, and transfection efficiency was verified and visualized in conjunction with GraphPad Prism software.

### 5.7. Proliferative Capacity of HCC-LM3 Cell Line After Knockdown of CDC25B Detected by EDU Method

The cell proliferation ability of the HCC-LM3 cell line after the knockdown of CDC25B and the HCC-LM3 cell line without the knockdown of CDC25B was compared by the EDU method. The cell images were observed and captured under a fluorescence microscope, and ImageJ software was used to process and analyze the results under an electron microscope, quantitatively analyze the images, calculate the proportion of positive cells, and visualize them jointly with GraphPad Prism software.

### 5.8. Immune-Infiltrating Cell Correlation Analysis

TIMER (https://cistrome.shinyapps.io/timer/) is a database-based web application capable of calculating the cellular infiltration levels of six major immune cell types including B cells, CD4+ T cells, CD8+ T cells, macrophages, neutrophils, and dendritic cells in the TME. TIMER is systematically calculated and collation. In this study, we investigated the potential correlation between CDC25B expression and immune cell infiltration in HCC patients using the data provided by the TIMER database.

### 5.9. Analysis of the Correlation Between CDC25B and Immune Checkpoints

TISIDB (http://cis.hku.hk/TISIDB/) is a portal for tumor-immune system interactions that integrates a variety of heterogeneous data types, and it shows the correlation between pan-cancer and immune checkpoints, including immunosuppressive and immunostimulatory sites. We investigated the potential correlation of the CDC25B gene with some tumor immune checkpoints in HCC.

### 5.10. Drug Sensitivity Analysis

The TCGA database was utilized to construct the expression matrix. The half-maximal IC50 of the drugs were calculated using R language software. This software package includes information on the effects of various drugs, and we investigated the relationship between the IC50 of common chemotherapeutic drugs used in the treatment of HCC patients and the expression of CDC25B. Box plots were plotted using the R software package “ggplot2,” and the difference was considered statistically significant at *p* < 0.05.

### 5.11. Mutation Analysis

We analyzed 11 genes including CDC25B and hub genes for mutations in HCC samples in the cbiopportal database.

## Data Availability

Original data will be available upon rational request.
